# Putting “sticky notes” on the electronic medical record to promote intra-hospital referral of hepatitis B and C virus-positive patients to hepatology specialists: an exploratory study

**DOI:** 10.1186/s12879-016-1765-y

**Published:** 2016-08-12

**Authors:** Hideki Fujii, Seiko Yamaguchi, Osamu Kurai, Masato Miyano, Wataru Ueda, Hiroko Oba, Tetsuya Aoki, Masaru Enomoto, Norifumi Kawada, Kiyotaka Okawa

**Affiliations:** 1 Department of Gastroenterology and Hepatology, Osaka City Juso Hospital, Nonaka-kita, Yodogawa, Osaka 532-0034 Japan; 2 Department of Hepatology, Graduate School of Medicine, Osaka City University, Abeno, Osaka 545-8585 Japan

**Keywords:** Hepatitis B virus, Hepatitis C virus, Screening, Intra-hospital referral

## Abstract

**Background:**

Currently, no system for appropriate intra-hospital collaboration regarding hepatitis virus positive individuals exists, even in medical institutions with hepatologists among their staff. The main objective of this study was to explore a simple alert system to promote the referral of patients with hepatitis B surface antigen (HBsAg)- or anti-hepatitis C virus (HCV) antibodies positivity to hepatologists through electronic medical records.

**Methods:**

Since April 2014 at Osaka City Juso Hospital, “sticky notes” have been put on the electronic medical records of patients newly diagnosed with HBsAg- or anti-HCV- antibodies positivity to recommend intra-hospital referral of those patients to specialists. In this study, we investigated the number of referrals to hepatologists before vs. after the introduction of this system (that is, in fiscal years 2013 [Period 1] and 2014 [Period 2], respectively), and the subsequent clinical courses of the patients.

**Results:**

The proportions of patients with HBsAg and anti-HCV antibody positivity did not show statistically significant differences between Period 1 and Period 2 (1.6 % [43/2,757] vs. 1.3 % [39/2,891], *p* = 0.58; and 5.8 % [156/2,674] vs. 5.3 % [147/2,790], *p* = 0.39, respectively). However, the referral proportions for patients with HBsAg- and anti-HCV antibody positivity were significantly higher in Period 2 (73 % [11/15] and 65 % [41/63], respectively) than in Period 1 (28 % [5/18] and 17 % [9/54]) (*p* = 0.009 and *p* < 0.001, respectively). Among patients who were referred to hepatologists, 2 HBsAg-positive and 4 anti-HCV antibody positive patients initiated antiviral treatment.

**Conclusion:**

Our simple electronic medical record based alert system effectively promoted intra-hospital referral of hepatitis virus-positive patients, who have been detected by screening tests, to hepatologists.

**Electronic supplementary material:**

The online version of this article (doi:10.1186/s12879-016-1765-y) contains supplementary material, which is available to authorized users.

## Background

Chronic infection with hepatitis B virus (HBV) and hepatitis C virus (HCV) is the major cause of end-stage liver diseases, including cirrhosis and hepatocellular carcinoma [1, 2]. However, disease progression and cancer development can be inhibited by antiviral treatment [3–6], especially with the recent advances in these antiviral treatments. Thus, referral of hepatitis virus- positive individuals to a hepatologist at least once in their lifetime is recommended [7]. Unfortunately, in many Western countries, even though patients are successfully screened, many do not receive confirmatory testing for HBV or HCV [8–11].

In Japan, although medical check-ups for HBV and HCV infection are conducted nationwide, not all those who are found to be positive for these viruses seek specialist medical attention or receive proper treatment from a hepatologist. Thus, the government is collaborating with two in-country civil society groups-the Japan Hepatitis Council and the Viral Hepatitis Research Foundation of Japan-to develop and implement its viral hepatitis prevention and control program [12]. In hospitals, testing for HBV and HCV is performed as part of daily practice, as a preoperative or pretransfusion screening test. However, the efficiency of intra-hospital collaboration with hepatologists in relation to the follow-up treatment of HBV- or HCV- positive individuals detected by screening tests remains unclear. Recently, Furukawa et al. reported on the current management practices for patients with hepatitis B surface antigen- (HBsAg-) and anti-HCV- antibody positivity in non-hepatology departments at Saga University Hospital [13]. The prevalence proportions of HBsAg- and anti-HCV antibody positive individuals were 1.9 and 5.6 %, respectively. However, 79 % of HBV- and 82 % of probable HCV- positive patients were not referred to hepatologists. To address this issue, a system to manage hepatitis virus- positive patients and to ensure that they receive specialist care should be established as soon as possible in medical institutions that have hepatologists among their staff.

We developed a simple alert system to promote the referral of HBsAg- or anti-HCV antibody positive patients to hepatology specialists through their electronic medical records. In this exploratory study, we investigated the changes in the number of referrals to hepatologists and in the distribution of patients referred after the introduction of the system, as well as the subsequent clinical courses of the patients following these referrals. Herein, we present our results. Further improvements needed for intra-hospital collaboration are discussed based on our findings.

## Methods

### Design

This was a single-center study of both inpatients and outpatients conducted in a secondary-care hospital. The investigation consisted of two parts, a retrospective investigation of the pre-intervention period (from April 1, 2013, to March 31, 2014; defined as “Period 1”) and a prospective investigation during the post-intervention period (from April 1, 2014, to March 31, 2015; defined as “Period 2”).

### Development of an alert system for promoting referrals of HBsAg- or anti-HCV antibody positive patients to a specialist department

Osaka City Juso Hospital is a community hospital with 263 beds (5 wards). Outpatient services are provided by 15 clinical departments, and approximately 500 patients visit the hospital daily. The personal identification numbers of newly diagnosed patients with HBsAg- or anti-HCV antibody positivity were sent from the hospital’s central laboratory to the principal investigator through the hospital intranet every Monday afternoon during Period 2. After receiving these numbers, the principal investigator entered the following comment, referred to as a “sticky note,” in the electronic medical records of any patient who had not been referred to a hepatologist : “This patient was found to be positive for HBsAg (or anti-HCV antibody) in this test. Please consider referring the patient to a hepatologist.” This was done every Thursday evening. Once “sticky noted,” every user of the medical record was forced to see this “sticky note”. The cut-off for the presence or absence of referral for the purposes of the study was set as 1 month after the medical records were “sticky noted.”

We compared the number of patients screened for either HBV or HCV in Period 2 with that in Period 1. The distribution across the referring departments of patients who were referred to a hepatologist from non-hepatology departments and the patients’ clinical courses after the referral was examined. The primary outcome of this study was the referral proportion to a hepatologist. The referral proportion was calculated as follows: referral proportion = number of patients referred to a hepatologist/ number of patients who attended a non-hepatologist appointment to receive a positive test result of HBsAg or anti-HCV antibody × 100.

The study protocol and informed consent procedure were approved by the Institutional Review Board at Osaka City Juso Hospital, and the study was conducted in accordance to the concepts of the Declaration of Helsinki. The Institutional Review Board gave permission to collect data without informed consent.

### Hepatitis virus testing

HBsAg was measured using the Architect HBsAg QT assay (Abbott Japan, Tokyo), and anti-HCV antibodies were measured using the Architect HCV assay (Abbott Japan, Tokyo). HBV-DNA was measured using COBAS TaqMan HBV Test v. 2.0 (Roche Diagnostics K.K., Tokyo, Japan), and HCV-RNA was measured using the COBAS TaqMan HCV Test v. 2.0 (Roche Diagnostics K.K.).

### Statistical analysis

Statistical analysis was conducted using JMP 11.0 software (SAS Institute, Inc., Cary, NC). Fisher’s exact probability test or the chi-square test was used for categorical factors. As no participant appeared in the study periods, these tests were considered valid and appropriate. Differences with *p* values <0.05 were considered statistically significant.

## Results

### Status of HBsAg and anti-HCV antibody testing

**Fig. 1:**
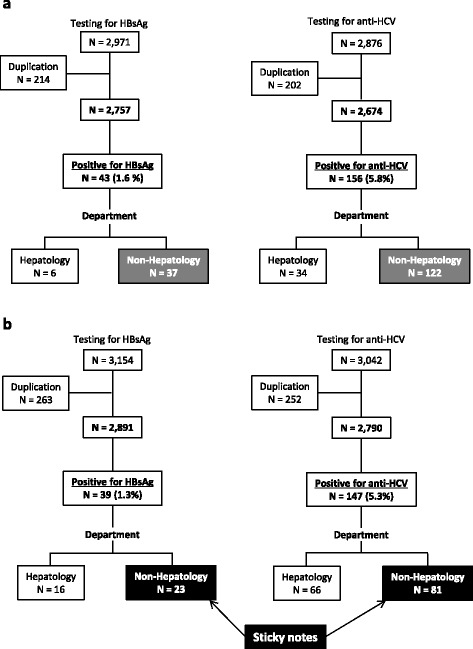
Flow chart of subjects who underwent screening tests for HBsAg or anti-HCV antibody. **a** in Period 1. **b** in Period 2. HBsAg, hepatitis B surface antigen; HCV, hepatitis C virus

In Period 1, of the 2,757 patients in whom HBsAg was measured, 43 (1.6 %) were found to be positive, and of the 2,674 patients who underwent anti-HCV antibody measurements, 156 (5.8 %) were found to be positive (Fig. 1a). In Period 2, of the 2,891 patients in whom HBsAg was measured, 39 (1.3 %) were found to be positive, and of the 2,790 patients who underwent anti-HCV antibody measurements, 147 (5.3 %) were found to be positive (Fig. 1b). The proportions of patients with HBsAg and anti-HCV antibody positivity did not show statistically significant differences between Period 1 and Period 2 (*p* = 0.58 and *p* = 0.39, respectively). We put “sticky notes” on the records for the 23 HBsAg- positive and 81 anti-HCV antibody positive patients who were not immediately referred to a hepatologist (Fig. 1b).

### Referral proportions of “sticky noted” patients to hepatologists

**Fig. 2:**
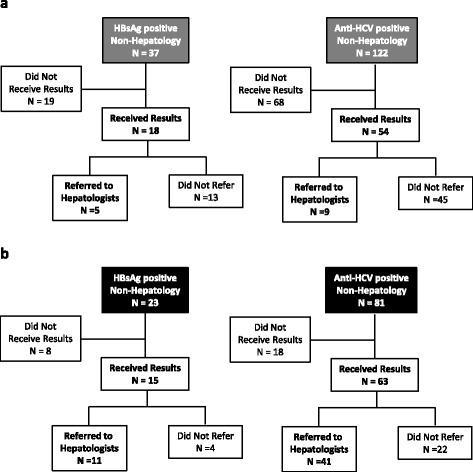
HBsAg or anti-HCV antibody positive subjects in non-hepatology departments referred to hepatologists (“sticky noted”). **a** in Period 1. **b** in Period 2. HBsAg, hepatitis B surface antigen; HCV, hepatitis C virus

Figure 2a, b show the flow charts of the study subjects who were found to be positive for HBsAg or anti-HCV antibody in non-hepatology departments and recommended to consult hepatologists (“sticky noted”) in Periods 1 and 2, respectively. Patients who did not attend the non-hepatologist appointment to receive the positive test results (ie those who were still unaware of their infection status) were excluded from the analysis of the referral proportions. The referral proportions for both HBsAg and anti-HCV antibody positive patients were significantly higher in Period 2 (73 % [11/15] and 65 % [41/63], respectively) than in Period 1 (28 % [5/18] and 17 % [9/54]) (*p* = 0.009 and *p* < 0.001, respectively).

### Distributions across referring non-hepatology departments of patients referred to a hepatologist during Period 2

Across the clinical departments of the hospital, the numbers of patients who were “sticky noted” in Period 2 were as follows: Orthopedics (*n* = 22), Respiratory Medicine (*n* = 20), Gastroenterology (*n* = 18), Urology (*n* = 11), Surgery (*n* = 11), Ophthalmology (*n* = 9), Obstetrics and Gynecology (*n* = 7), Diabetic Medicine (*n* = 4), and Pediatrics (*n* = 2). Of these, the numbers of patients who were actually referred to hepatologists were as follows: Gastroenterology (*n* = 13 [72 %]), Respiratory Medicine (*n* = 11 [55 %]), Orthopedics (*n* = 9 [41 %]), Obstetrics and Gynecology (*n* = 5 [71 %]), Urology (*n* = 4 [36 %]), Surgery (*n* = 3 [27 %]), Ophthalmology (*n* = 3 [33 %]), Diabetic Medicine (*n* = 2 [50 %]), and Pediatrics (*n* = 2 [100 %]) (Additional file 1: Figure S1).

### Outcomes of patients referred to hepatologists

As shown in Fig. 2a, b, of the 23 HBsAg positive patients who were “sticky noted,” 11 ultimately consulted a hepatologist; of these, two started nucleoside analogue treatment, while nine were found to be inactive HBV- positive patients who required only periodic follow- up. Of the 81 anti-HCV antibody- positive patients who were “sticky noted,” 41 ultimately consulted a hepatologist; of these, four started interferon-based or first-generation interferon-free treatment, ten were listed as being considered for next-generation antiviral treatment, and 27 were negative for HCV-RNA. The clinical backgrounds of the six patients who received antiviral treatment are shown in Additional file 2: Table S1.

## Discussion

We developed a simple alert system to refer HBsAg- or anti-HCV antibody- positive patients to hepatologists through electronic medical records. The main finding of this study was that our system could increase the number of referrals of hepatitis virus-positive patients detected by screening tests to hepatologists. Similarly, Shimomura et al. recently reported that the implementation of a fully automated reporting system relying on electronic medical records at Okayama University Hospital also increased the number of referrals of HBsAg- or anti-HCV antibody- positive patients to the Hepatology department [14], indicating that such alert system may have the potential to promote intra-hospital referrals in other areas as well. For example, HbA1C is widely used as a screening tool for diabetes mellitus [15], and thus may help promote intra-hospital referrals of high-risk diabetic patients to specialists.

Electronic medical records have the potential to provide substantial benefits to physicians, clinical practices, and health care organizations [16], with the system optimization process being defined as the continuing effort to help users maximize their proficiency in the use of the system [17]. Our alert system has certain important advantages. First, this system is easy to operate. Although it is a manual system, it requires little effort to implement. We created approximately 100 “sticky notes” on the patients’ electronic medical records over the course of 1 year; this is equivalent to 1 “sticky note” every 3 days, and it takes only a minute or two to place such a “sticky note”. This level of additional workload seems to be acceptable even for busy medical staff. Next, this system is free to use, and it is easy to optimize the existing electronic medical record- based systems. Moreover, in relatively small community hospitals like ours, where care decisions are generally made in only a short time, it is easy to familiarize the staff with this system.

In Japan, the numbers of anti-HCV antibody- and HBsAg- positive individuals are estimated to be 1.5–2 million and 1.3–1.5 million, respectively. However, Tanaka et al. estimated that approximately 800,000 anti-HCV antibody- positive and 900,000 HBsAg-positive individuals are not aware that they are infected [18]. Moreover, the national Ministry of Health, Labour, and Welfare Ministerial Notification No. 160 (released on May 16, 2011) requested that medical institutions properly explain the results of hepatitis virus testing to the individuals tested. Although antiviral treatment has advanced substantially, if infected patients screened on various occasions do not consult a hepatologist and receive proper treatment, they will not benefit from the recent advances made in antiviral treatment. Additionally, even when the viruses are eliminated, cancer may sometimes develop, especially in older patients and in those with advanced fibrosis. Thus, unless proper follow-up is continued over the lifetime of the patients, reductions in liver-related deaths cannot ultimately be achieved.

Even with “sticky notes”, the referral proportion was still comparably low in some departments in our hospital (<50 % referral proportions in the Orthopedics, Urology, Surgery, and Ophthalmology Departments). The reason for this low proportion is unclear, but it is probable that the referral proportion varies with the varying awareness of the physicians regarding appropriate responses for patients with hepatitis virus infection. It is also necessary for hepatologists to make efforts to inform physicians in non-hepatology departments about the recent advances in antiviral treatments at any opportunity, for example at conferences.

Some limitations of this study should be mentioned. First, the referral proportion did not directly reflect the number of patients who subsequently received care for HBV or HCV infection. One reason is that some patients referred to hepatologists have already received care for HBV or HCV in other hospitals. Second, this was a single-centre study conducted in a secondary care hospital. A manual “sticky note” system as utilized in this study may be of little relevance or clinical assistance in tertiary care hospitals or public health settings. Third, some confounders, such as staff education, awareness of clinical trials, and availability of antiviral therapy for HBV and HCV may have affected the referral proportions.

## Conclusions

In conclusion, the simple, electronic medical record- based alert system described herein was found to be effective for promoting intra-hospital referrals of hepatitis virus positive patients detected by screening tests to hepatologists. In addition, improved knowledge and awareness of HBV and HCV infection among healthcare service providers appear important in terms of enabling intra-hospital collaborations and specialist referrals.

## Abbreviations

HBsAg, hepatitis B surface antigen; HBV, hepatitis B virus; HCV, hepatitis C virus

## Additional files

Additional file 1: Distribution of patients in non-hepatology departments who were referred to hepatologists in Period 2. (PPTX 51 kb) Additional file 2: Detailed data about the patients (*n* = 6) who received anti-viral treatment in Period 2. (PPTX 54 kb)
