# Supplementary material for: Putting “sticky notes” on the electronic medical record to promote intra-hospital referral of hepatitis B and C virus-positive patients to hepatology specialists: an exploratory study
Source: BMC Infect Dis. 2016 Aug 12;16:410. doi: 10.1186/s12879-016-1765-y (PMC4983008; doi:10.1186/s12879-016-1765-y)
Supplement: Additional file 1: — Distribution of patients in non-hepatology departments who were referred to hepatologists in Period 2. (PPTX 51 kb) [file 12879_2016_1765_MOESM1_ESM.pptx]

## Slide 1
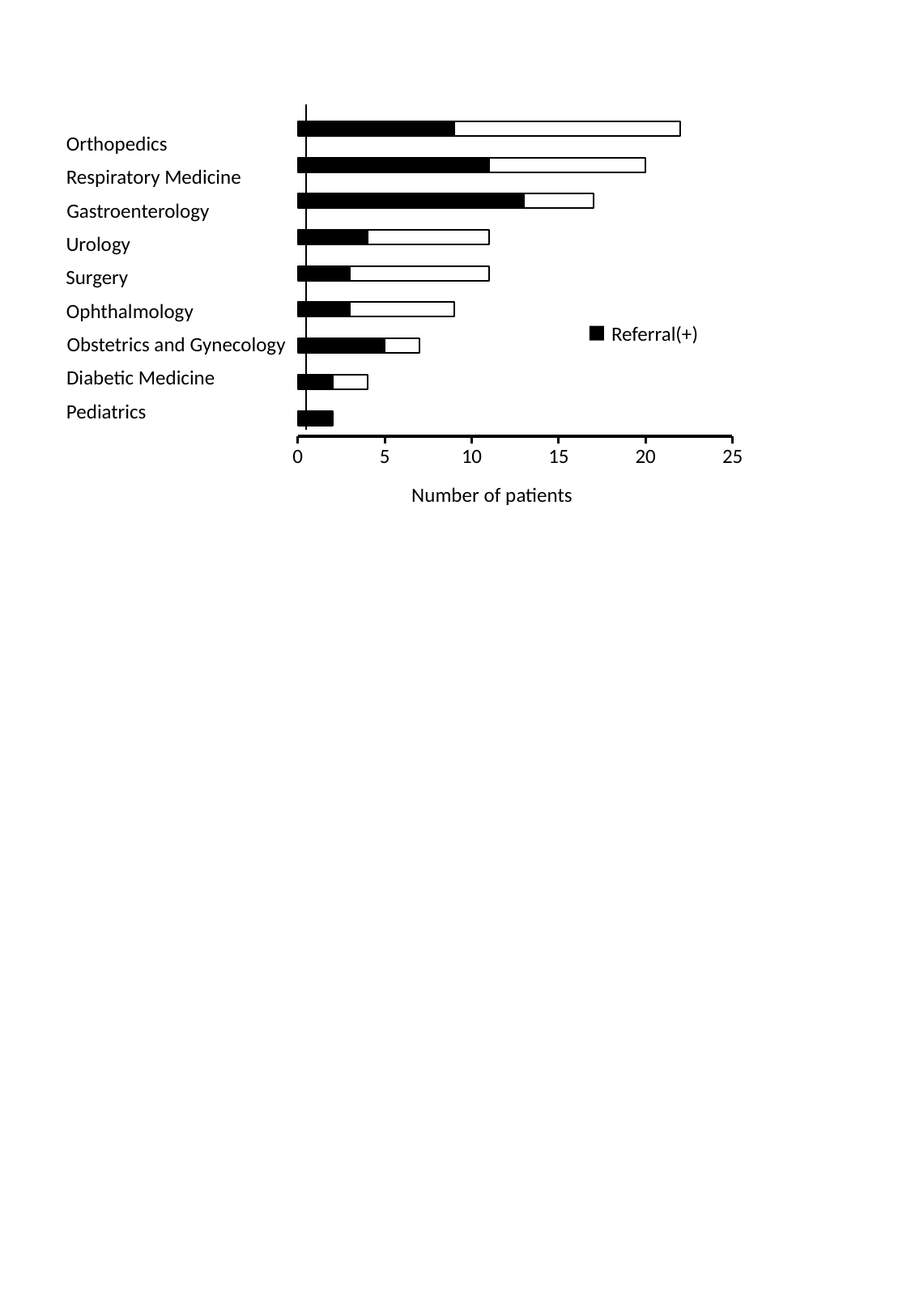

### Chart
| Category | 紹介(+) | 紹介(-) |
|---|---|---|
| 小児 | 2.0 | 0.0 |
| 糖内 | 2.0 | 2.0 |
| 産婦 | 5.0 | 2.0 |
| 眼科 | 3.0 | 6.0 |
| 外科 | 3.0 | 8.0 |
| 泌尿 | 4.0 | 7.0 |
| 消内 | 13.0 | 4.0 |
| 呼内 | 11.0 | 9.0 |
| 整形 | 9.0 | 13.0 |Orthopedics
Respiratory Medicine
Gastroenterology
Urology
Surgery
Ophthalmology
Obstetrics and Gynecology
Diabetic Medicine
Pediatrics
Referral(+)
Number of patients
