# Supplementary material for: Putting “sticky notes” on the electronic medical record to promote intra-hospital referral of hepatitis B and C virus-positive patients to hepatology specialists: an exploratory study
Source: BMC Infect Dis. 2016 Aug 12;16:410. doi: 10.1186/s12879-016-1765-y (PMC4983008; doi:10.1186/s12879-016-1765-y)
Supplement: Additional file 2: — Detailed data about the patients (n = 6) who received anti-viral treatment in Period 2. (PPTX 54 kb) [file 12879_2016_1765_MOESM2_ESM.pptx]

## Slide 1
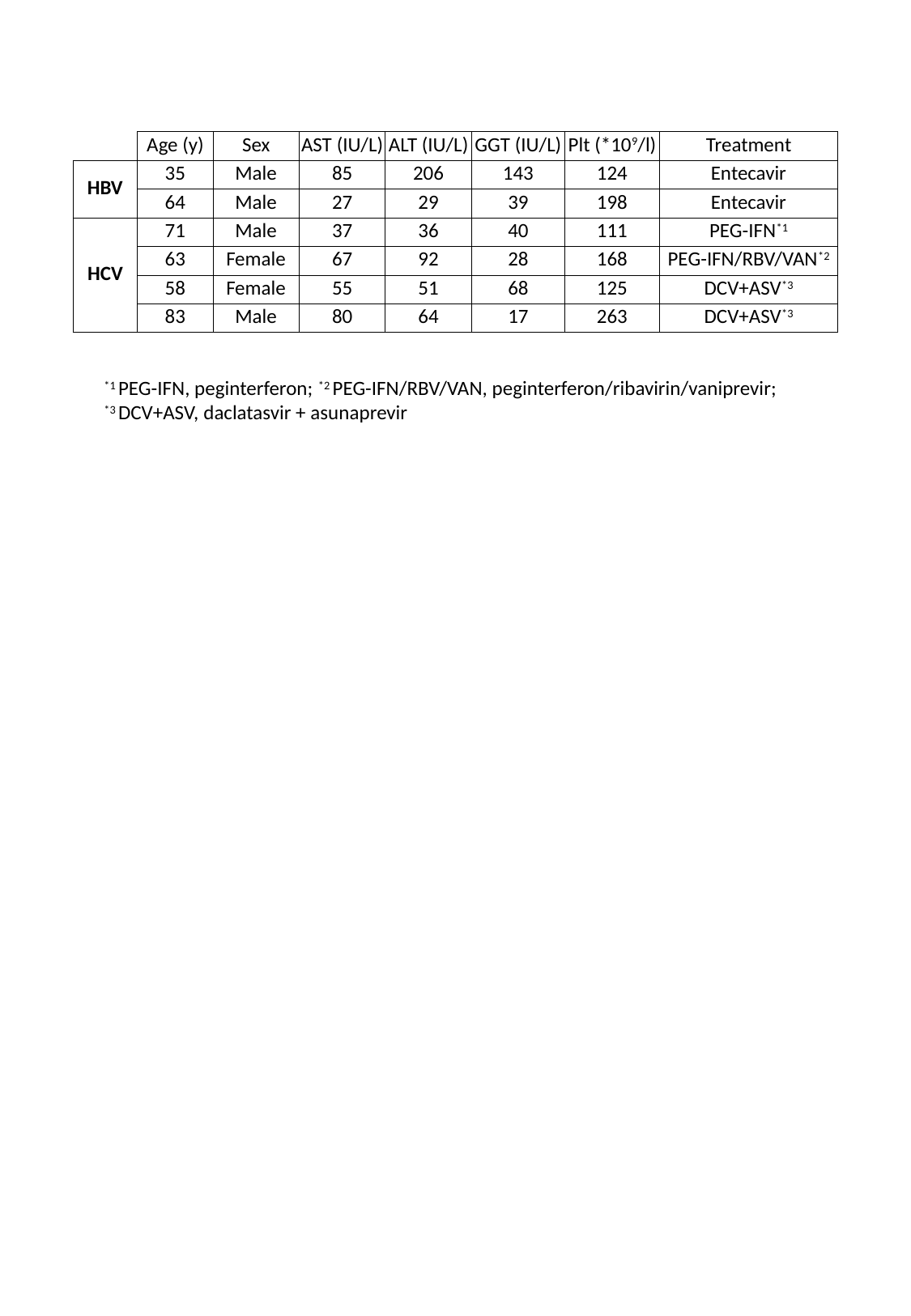

| | Age (y) | Sex | AST (IU/L) | ALT (IU/L) | GGT (IU/L) | Plt (\*109/l) | Treatment |
| --- | --- | --- | --- | --- | --- | --- | --- |
| HBV | 35 | Male | 85 | 206 | 143 | 124 | Entecavir |
| | 64 | Male | 27 | 29 | 39 | 198 | Entecavir |
| HCV | 71 | Male | 37 | 36 | 40 | 111 | PEG-IFN\*1 |
| | 63 | Female | 67 | 92 | 28 | 168 | PEG-IFN/RBV/VAN\*2 |
| | 58 | Female | 55 | 51 | 68 | 125 | DCV+ASV\*3 |
| | 83 | Male | 80 | 64 | 17 | 263 | DCV+ASV\*3 |
*1 PEG-IFN, peginterferon; *2 PEG-IFN/RBV/VAN, peginterferon/ribavirin/vaniprevir; *3 DCV+ASV, daclatasvir + asunaprevir
